# Behavior of a Competitive System of Second-Order Difference Equations

**DOI:** 10.1155/2014/283982

**Published:** 2014-05-15

**Authors:** Q. Din, T. F. Ibrahim, K. A. Khan

**Affiliations:** ^1^Department of Mathematics, Faculty of Basic and Applied Sciences, University of Poonch Rawalakot, Rawalakot 12350, Pakistan; ^2^Department of Mathematics, Faculty of Sciences and Arts (S.A.), King Khalid University, Abha, Sarat Abida 61914, Saudi Arabia; ^3^Department of Mathematics, Faculty of Science, Mansoura University, Mansoura 35516, Egypt; ^4^Department of Mathematics, University of Sargodha, Sargodha 40100, Pakistan

## Abstract

We study the boundedness and persistence, existence, and uniqueness of positive equilibrium, local and global behavior of positive equilibrium point, and rate of convergence of positive solutions of the following system of rational difference equations: *x*_*n*+1_ = *(α*_1_ + *β*_1_ *x*_*n*−1_)/*(a*_1_ + *b*_1_ *y*_*n*_), *y*_*n*+1_ = *(α*_2_ + *β*_2_ *y*_*n*−1_)/*(a*_2_ + *b*_2_ *x*_*n*_), where the parameters *α*_*i*_, *β*_*i*_, *a*_*i*_, and *b*_*i*_ for *i* ∈ {1,2} and initial conditions *x*_0_, *x*_−1_, *y*_0_, and *y*_−1_ are positive real numbers. Some numerical examples are given to verify our theoretical results.

## 1. Introduction

Systems of nonlinear difference equations of higher order are of paramount importance in applications. Such equations also appear naturally as discrete analogues and as numerical solutions of systems differential and delay differential equations which model diverse phenomena in biology, ecology, physiology, physics, engineering, and economics. For applications and basic theory of rational difference equations, we refer to [1–3]. In [4–10], applications of difference equations in mathematical biology are given. Nonlinear difference equations can be used in population models [11–17]. It is very interesting to investigate the behavior of solutions of a system of nonlinear difference equations and to discuss the local asymptotic stability of their equilibrium points.

Gibbons et al. [18] investigated the qualitative behavior of the following second-order rational difference equation:

(1) $$\begin{array}{c} x_{n+1}=\frac{\alpha +\beta x_{n-1}}{\gamma +x_{n}}. \end{array}$$

Motivated by the above study, our aim in this paper is to investigate the qualitative behavior of positive solutions of the following second-order system of rational difference equations:

(2) $$\begin{array}{c} x_{n+1}=\frac{\alpha_{1}+\beta_{1}x_{n-1}}{a_{1}+b_{1}y_{n}},\;\;y_{n+1}=\frac{\alpha_{2}+\beta_{2}y_{n-1}}{a_{2}+b_{2}x_{n}}, \end{array}$$

where the parameters *α*_*i*_, *β*_*i*_, *a*_*i*_, and *b*_*i*_ for *i* ∈ {1, 2} and initial conditions *x*_0_, *x*_−1_, *y*_0_, and *y*_−1_ are positive real numbers.

More precisely, we investigate the boundedness character, persistence, existence, and uniqueness of positive steady state, local asymptotic stability, and global behavior of unique positive equilibrium point and rate of convergence of positive solutions of system (2) which converge to its unique positive equilibrium point.

## 2. Boundedness and Persistence

The following theorem shows the boundedness and persistence of every positive solution of system (2).

**Theorem 1**

Assume that *β*_1_ < *a*_1_ and *β*_2_ < *a*_2_; then every positive solution {(*x*_*n*_, *y*_*n*_)} of system (2) is bounded and persists.

**Proof**

For any positive solution {(*x*_*n*_, *y*_*n*_)} of system (2), one has

(3) xn+1≤A1+B1xn−1,  yn+1≤A2+B2yn−1,n=0,1,2,…,

where *A*_*i*_ = *α*_*i*_/*a*_*i*_ and *B*_*i*_ = *β*_*i*_/*a*_*i*_ for *i* ∈ {1, 2}. Consider the following linear difference equations:

(4) $$\begin{array}{c} u_{n+1}=A_{1}+B_{1}u_{n-1},\;n=0,1,2,\ldots ,\\ v_{n+1}=A_{2}+B_{2}v_{n-1},\;n=0,1,2,\ldots . \end{array}$$

Obviously, solutions of these second-order nonhomogeneous difference equations are given by

(5) $$\begin{array}{c} u_{n}=\frac{A_{1}}{1-B_{1}}+c_{1}B_{1}^{n/2}+c_{2}{\left(-\sqrt{B_{1}}\right)}^{n},\;n=1,2,\ldots ,\\ v_{n}=\frac{A_{2}}{1-B_{2}}+c_{3}B_{2}^{n/2}+c_{4}{\left(-\sqrt{B_{2}}\right)}^{n},\;n=1,2,\ldots , \end{array}$$

where *c*_*i*_ for *i* ∈ {1, 2, 3, 4} depend upon initial conditions *u*_−1_, *u*_0_, *v*_−1_, and *v*_0_. Assume that *β*_1_ < *a*_1_ and *β*_2_ < *a*_2_; then the sequences {*u*_*n*_} and {*v*_*n*_} are bounded. Suppose that *u*_−1_ = *x*_−1_, *u*_0_ = *x*_0_, *v*_−1_ = *y*_−1_, and *v*_0_ = *y*_0_; then by comparison we have

(6) xn≤α1a1−β1=U1,  yn≤α2a2−β2=U2,n=1,2,….

Furthermore, from system (2) and (6) we obtain that

(7) $$\begin{array}{c} x_{n+1}\geq \frac{\alpha_{1}}{a_{1}+b_{1}y_{n}}\geq \frac{\alpha_{1}\left(a_{2}-\beta_{2}\right)}{a_{1}\left(a_{2}-\beta_{2}\right)+b_{1}\alpha_{2}}=L_{1},\\ y_{n+1}\geq \frac{\alpha_{2}}{a_{2}+b_{2}x_{n}}\geq \frac{\alpha_{2}\left(a_{1}-\beta_{1}\right)}{a_{2}\left(a_{1}-\beta_{1}\right)+b_{2}\alpha_{1}}=L_{2}. \end{array}$$

From (6) and (7), it follows that

(8) $$\begin{array}{c} L_{1}\leq x_{n}\leq U_{1},\;\;L_{2}\leq y_{n}\leq U_{2},\;n=1,2,\ldots . \end{array}$$

Hence, theorem is proved.

**Lemma 2**

Let {(*x*_*n*_, *y*_*n*_)} be a positive solution of system (2). Then, [*L*_1_, *U*_1_] × [*L*_2_, *U*_2_] is invariant set for system (2).

**Proof**

The proof follows by induction.

## 3. Stability Analysis

Let us consider fourth-dimensional discrete dynamical system of the following form:

(9) xn+1=f(xn,xn−1,yn,yn−1),yn+1=g(xn,xn−1,yn,yn−1),n=0,1,…,

where *f* : *I* ^2^ × *J* ^2^ → *I* and *g* : *I* ^2^ × *J* ^2^ → *J* are continuously differentiable functions and *I*, *J* are some intervals of real numbers. Furthermore, a solution {(*x*_*n*_,*y*_*n*_)}_*n*=−1_^*∞*^ of system (9) is uniquely determined by initial conditions (*x*_*i*_, *y*_*i*_) ∈ *I* × *J* for *i* ∈ {−1,0}. Along with system (9), we consider the corresponding vector map *F* = (*f*, *x*_*n*_, *g*, *y*_*n*_). An equilibrium point of (9) is a point $\left(\bar{x},\bar{y}\right)$ that satisfies

(10) $$\begin{array}{c} \bar{x}=f\left(\bar{x},\bar{x},\bar{y},\bar{y}\right),\\ \bar{y}=g\left(\bar{x},\bar{x},\bar{y},\bar{y}\right). \end{array}$$

The point $\left(\bar{x},\bar{y}\right)$ is also called a fixed point of the vector map *F*.

**Definition 3**

Let $\left(\bar{x},\bar{y}\right)$ be an equilibrium point of the system (9).

(i) An equilibrium point $\left(\bar{x},\bar{y}\right)$ is said to be stable if for every *ε* > 0 there exists *δ* > 0 such that, for every initial condition (*x*_*i*_, *y*_*i*_), *i* ∈ {−1,0} if $||\sum_{i=-1}^{0}\left(x_{i},y_{i}\right)-\left(\bar{x},\bar{y}\right)||<\delta$ implies that $||\left(x_{n},y_{n}\right)-\left(\bar{x},\bar{y}\right)||<\varepsilon$ for all *n* > 0, where ||·|| is usual Euclidian norm in *R* ^2^.
(ii) An equilibrium point $\left(\bar{x},\bar{y}\right)$ is said to be unstable if it is not stable.
(iii) An equilibrium point $\left(\bar{x},\bar{y}\right)$ is said to be asymptotically stable if there exists *η* > 0 such that
(11) $$\begin{array}{c} ||\overset{0}{\underset{i=-1}{\sum }}\left(x_{i},y_{i}\right)-\left(\bar{x},\bar{y}\right)||<\eta ,\\ \left(x_{n},y_{n}\right)⟶\left(\bar{x},\bar{y}\right)\;\text{as}\;n⟶\infty . \end{array}$$
(iv) An equilibrium point $\left(\bar{x},\bar{y}\right)$ is called global attractor if $\left(x_{n},y_{n}\right)\to \left(\bar{x},\bar{y}\right)$ as *n* → *∞*.
(v) An equilibrium point $\left(\bar{x},\bar{y}\right)$ is called asymptotic global attractor if it is a global attractor and stable.

**Definition 4**

Let $\left(\bar{x},\bar{y}\right)$ be an equilibrium point of a map *F* = (*f*, *x*_*n*_, *g*, *y*_*n*_), where *f* and *g* are continuously differentiable functions at $\left(\bar{x},\bar{y}\right)$ . The linearized system of (9) about the equilibrium point $\left(\bar{x},\bar{y}\right)$ is

(12) $$\begin{array}{c} X_{n+1}=F\left(X_{n}\right)=F_{J}X_{n}, \end{array}$$

where $X_{n}=\left(\begin{array}{c} x_{n}\\ y_{n}\\ x_{n-1}\\ y_{n-1} \end{array}\right)$ and *F*_*J*_ is Jacobian matrix of system (9) about the equilibrium point $\left(\bar{x},\bar{y}\right)$ .

To construct the corresponding linearized form of system (2) we consider the following transformation:

(13) $$\begin{array}{c} \left(x_{n},y_{n},x_{n-1},y_{n-1}\right)⟼\left(f,g,f_{1},g_{1}\right), \end{array}$$

where *f* = *x*_*n*+1_, *g* = *y*_*n*+1_, *f*_1_ = *x*_*n*_, and *g*_1_ = *y*_*n*_. The linearized system of (2) about $\left(\bar{x},\bar{y}\right)$ is given by

(14) $$\begin{array}{c} Z_{n+1}=F_{J}\left(\bar{x},\bar{y}\right)Z_{n}, \end{array}$$

where $Z_{n}=\left(\begin{array}{c} x_{n}\\ y_{n}\\ x_{n-1}\\ y_{n-1} \end{array}\right)$ and the Jacobian matrix about the fixed point $\left(\bar{x},\bar{y}\right)$ under the transformation (13) is given by

(15) FJ(x¯,y¯)=(0−b1x¯a1+b1y¯β1a1+b1y¯0−b2y¯a2+b2x¯00β2a2+b2x¯10000100).

**Lemma 5**

Assume that *X*_*n*+1_ = *F*(*X*_*n*_), *n* = 0,1,…, is a system of difference equations such that $\bar{X}$ is a fixed point of *F*. If all eigenvalues of the Jacobian matrix *J*_*F*_ about $\bar{X}$ lie inside the open unit disk |*λ*| < 1, then $\bar{X}$ is locally asymptotically stable. If one of them has a modulus greater than one, then $\bar{X}$ is unstable.

The following theorem shows the existence and uniqueness of positive equilibrium point of system (2).

**Theorem 6**

Assume that *β*_1_ < *a*_1_ and *β*_2_ < *a*_2_; then there exists unique positive equilibrium point of system (2) in [*L*_1_, *U*_1_] × [*L*_2_, *U*_2_], if the following condition is satisfied:

(16) α1α2b1b2 <(a1a2+b2(a1−β1)L1−a1β2−a2β1+α2b1+β1β2)2.

**Proof**

Consider the following system of equations:

(17) $$\begin{array}{c} x=\frac{\alpha_{1}+\beta_{1}x}{a_{1}+b_{1}y},\;\;y=\frac{\alpha_{2}+\beta_{2}y}{a_{2}+b_{2}x}. \end{array}$$

Assume that (*x*, *y*) ∈ [*L*_1_, *U*_1_] × [*L*_2_, *U*_2_]; then it follows from (17) that

(18) $$\begin{array}{c} x=\frac{\alpha_{1}}{a_{1}-\beta_{1}+b_{1}y},\;\;y=\frac{\alpha_{2}}{a_{2}-\beta_{2}+b_{2}x}. \end{array}$$

Take

(19) $$\begin{array}{c} F\left(x\right)=\frac{\alpha_{1}}{a_{1}-\beta_{1}+b_{1}f\left(x\right)}-x, \end{array}$$

where *f*(*x*) = *α*_2_/(*a*_2_ − *β*_2_ + *b*_2_ *x*) and *x* ∈ [*L*_1_, *U*_1_]. Then, we obtain that

(20) $$\begin{array}{c} f\left(L_{1}\right)=\frac{\alpha_{2}}{a_{2}-\beta_{2}}\left(\frac{a_{1}\left(a_{2}-\beta_{2}\right)+b_{1}\alpha_{2}}{a_{1}\left(a_{2}-\beta_{2}\right)+b_{1}\alpha_{2}+b_{2}\alpha_{1}}\right)<\frac{\alpha_{2}}{a_{2}-\beta_{2}}. \end{array}$$

Hence, it follows that

(21) F(L1)=α1a1−β1+b1f(L1)−L1>α1(a2−β2)(a1−β1)(a2−β2)+b1α2−L1=α1(a2−β2)(a1−β1)(a2−β2)+b1α2 −α1(a2−β2)a1(a2−β2)+b1α2>0.

Furthermore,

(22) F(U1)=α1a1−β1+b1f(U1)−U1=α1a1−β1((a2−β2)(a1−β1)+α1b2(a2−β2)(a1−β1)+α1b2+b1α2−1)<0.

Hence, *F*(*x*) = 0 has at least one positive solution in [*L*_1_, *U*_1_].

Furthermore, assume that condition (16) is satisfied; then one has

(23) F′(x) =(α1α2b1b2((a1a2+a1b2x−a1β2−a2β1  +α2b1+β1β2−β1b2x)2)−1)−1 ≤(α1α2b1b2((a1a2+b2(a1−β1)L1−a1β2−a2β1+α2b1+β1β2)2)−1)−1 <0.

Hence, *F*(*x*) = 0 has a unique positive solution in [*L*_1_, *U*_1_]. The proof is therefore completed.

**Theorem 7**

The unique positive equilibrium point $\left(\bar{x},\bar{y}\right)$ of system (2) is locally asymptotically stable if  *b*_1_ *b*_2_ *U*_1_ *U*_2_ + *β*_1_ *β*_2_ + *β*_1_(*a*_2_ + *b*_2_ *L*_1_) + *β*_2_(*a*_1_ + *b*_1_ *L*_2_) < (*a*_1_ + *b*_1_ *L*_2_)(*a*_2_ + *b*_2_ *L*_1_).

**Proof**

The characteristic polynomial of Jacobian matrix $F_{J}(\bar{x},\bar{y})$ about $\left(\bar{x},\bar{y}\right)$ is given by

(24) P(λ) =λ4−(b1b2x¯y¯(a1+b1y¯)(a2+b2x¯)+β1a1+b1y¯+β2a2+b2x¯)λ2  +β1β2(a1+b1y¯)(a2+b2x¯).

Let Φ(*λ*) = *λ* ^4^ and $\Psi \left(\lambda \right)=\left(\left(b_{1}b_{2}\bar{x}\bar{y}/\left(a_{1}+b_{1}\bar{y})(a_{2}+b_{2}\bar{x}\right)\right)+\left(\beta_{1}/\left(a_{1}+b_{1}\bar{y}\right)\right)+\left(\beta_{2}/\left(a_{2}+b_{2}\bar{x}\right)\right)\right)\lambda^{2}-\left(\beta_{1}\beta_{2}/\left(a_{1}+b_{1}\bar{y})(a_{2}+b_{2}\bar{x}\right)\right)$ . Assume that *b*_1_ *b*_2_ *U*_1_ *U*_2_ + *β*_1_ *β*_2_ + *β*_1_(*a*_2_ + *b*_2_ *L*_1_) + *β*_2_(*a*_1_ + *b*_1_ *L*_2_) < (*a*_1_ + *b*_1_ *L*_2_)(*a*_2_ + *b*_2_ *L*_1_) and |*λ*| = 1; then one has

(25) |Ψ(λ)| <(b1b2x¯y¯(a1+b1y¯)(a2+b2x¯)+β1a1+b1y¯+β2a2+b2x¯)  +β1β2(a1+b1y¯)(a2+b2x¯) <b1b2U1U2(a1+b1L2)(a2+b2L1)+β1a1+b1L2  +β2a2+b2L1+β1β2(a1+b1L2)(a2+b2L1) =b1b2U1U2+β1β2+β1(a2+b2L1)+β2(a1+b1L2)(a1+b1L2)(a2+b2L1) <1.

Then, by Rouche's Theorem, Φ(*λ*) and Φ(*λ*) − Ψ(*λ*) have the same number of zeroes in an open unit disk |*λ*| < 1. Hence, all the roots of (24) satisfy |*λ*| < 1, and it follows from Lemma 5 that the unique positive equilibrium point $\left(\bar{x},\bar{y}\right)$ of the system (2) is locally asymptotically stable.

Arguing as in [2], we have following result for global behavior of (2).

**Lemma 8**

Assume that *f* : (0, *∞*)×(0, *∞*)→(0, *∞*) and *g* : (0, *∞*)×(0, *∞*)→(0, *∞*) are continuous functions and *a*, *b*, *c*, and *d* are positive real numbers with *a* < *b*, *c* < *d*. Moreover, suppose that *f* : [*a*, *b*]×[*c*, *d*]→[*a*, *b*] and *g* : [*a*, *b*]×[*c*, *d*]→[*c*, *d*] such that following conditions are satisfied:

- *f*(*x*, *y*) is increasing in *x* and decreasing in *y*, and *g*(*x*, *y*) is decreasing in *x* and increasing in *y*;
- let *m*_1_,  *M*_1_,  *m*_2_, and *M*_2_ be real numbers such that *m*_1_ = *f*(*m*_1_, *M*_2_), *M*_1_ = *f*(*M*_1_, *m*_2_), *m*_2_ = *g*(*M*_1_, *m*_2_), and *M*_2_ = *g*(*m*_1_, *M*_2_); then *m*_1_ = *M*_1_ and *m*_2_ = *M*_2_.

Then, the system of difference equations *x*_*n*+1_ = *f*(*x*_*n*−1_, *y*_*n*_), *y*_*n*+1_ = *g*(*x*_*n*_, *y*_*n*−1_) has a unique positive equilibrium point $\left(\bar{x},\bar{y}\right)$ such that $\lim_{n\to \infty }(x_{n},y_{n})=(\bar{x},\bar{y})$ .

**Theorem 9**

The unique positive equilibrium point of system (2) is global attractor if (*a*_1_ − *β*_1_ + *b*_1_ *L*_2_)^2^(*a*_2_ − *β*_2_ + *b*_1_ *L*_1_)^2^ > *α*_1_ *α*_2_ *b*_1_ *b*_2_.

**Proof**

Let *f*(*x*, *y*) = (*α*_1_ + *β*_1_ *x*)/(*a*_1_ + *b*_1_ *y*) and *g*(*x*, *y*) = (*α*_2_ + *β*_2_ *y*)/(*a*_2_ + *b*_2_ *x*). Then, it is easy to see that *f*(*x*, *y*) is increasing in *x* and decreasing in *y*. Moreover, *g*(*x*, *y*) is decreasing in *x* and increasing in *y*. Let (*m*_1_, *M*_1_, *m*_2_, *M*_2_) be a solution of the system

(26) $$\begin{array}{c} m_{1}=f\left(m_{1},M_{2}\right),\;\;M_{1}=f\left(M_{1},m_{2}\right),\\ m_{2}=g\left(M_{1},m_{2}\right),\;\;M_{2}=g\left(m_{1},M_{2}\right). \end{array}$$

Then, one has

(27) $$\begin{array}{c} m_{1}=\frac{\alpha_{1}+\beta_{1}m_{1}}{a_{1}+b_{1}M_{2}},\;\;M_{1}=\frac{\alpha_{1}+\beta_{1}M_{1}}{a_{1}+b_{1}m_{2}},\\ m_{2}=\frac{\alpha_{2}+\beta_{2}m_{2}}{a_{2}+b_{2}M_{1}},\;\;M_{2}=\frac{\alpha_{2}+\beta_{2}M_{2}}{a_{2}+b_{2}m_{1}}. \end{array}$$

Furthermore, we have

(28) $$\begin{array}{c} L_{1}\leq m_{1},\;\;M_{1}\leq U_{1},\\ L_{2}\leq m_{2},\;\;M_{2}\leq U_{2}. \end{array}$$

From (27), it follows that

(29) $$\begin{array}{c} m_{1}=\frac{\alpha_{1}}{a_{1}-\beta_{1}+b_{1}M_{2}},\;\;M_{1}=\frac{\alpha_{1}}{a_{1}-\beta_{1}+b_{1}m_{2}}, \end{array}$$

(30) $$\begin{array}{c} m_{2}=\frac{\alpha_{2}}{a_{2}-\beta_{2}+b_{2}M_{1}},\;\;M_{2}=\frac{\alpha_{2}}{a_{2}-\beta_{2}+b_{2}m_{1}}. \end{array}$$

On subtracting (29), one has

(31) M1−m1 =α1(1a1−β1+b1m2−1a1−β1+b1M2) =α1b1(M2−m2)(a1−β1+b1m2)(a1−β1+b1M2) ≤α1b1(M2−m2)(a1−β1+b1L2)2.

Similarly, from (30), we obtain

(32) $$\begin{array}{c} M_{2}-m_{2}\leq \frac{\alpha_{2}b_{2}\left(M_{1}-m_{1}\right)}{{\left(a_{2}-\beta_{2}+b_{1}L_{1}\right)}^{2}}. \end{array}$$

Furthermore, from (31) and (32), we obtain

(33) $$\begin{array}{c} \left(K-\alpha_{1}\alpha_{2}b_{1}b_{2}\right)\left(M_{1}-m_{1}\right)\leq 0, \end{array}$$

where *K* = (*a*_1_ − *β*_1_ + *b*_1_ *L*_2_)^2^(*a*_2_ − *β*_2_ + *b*_1_ *L*_1_)^2^. Finally, from (33), it follows that *m*_1_ = *M*_1_. Similarly, it is easy to see that *m*_2_ = *M*_2_.

**Lemma 10**

Under the conditions of Theorems 7 and 9 the unique positive equilibrium of (2) is globally asymptotically stable.

## 4. Rate of Convergence

In this section, we will determine the rate of convergence of a solution that converges to the unique positive equilibrium point of the system (2).

The following result gives the rate of convergence of solutions of a system of difference equations:

(34) $$\begin{array}{c} X_{n+1}=\left(A+B\left(n\right)\right)X_{n}, \end{array}$$

where *X*_*n*_ is an *m*-dimensional vector, *A* ∈ *C* ^*m*×*m*^ is a constant matrix, and *B* : *Z* ^+^ → *C* ^*m*×*m*^ is a matrix function satisfying

(35) $$\begin{array}{c} ||B\left(n\right)||⟶0 \end{array}$$

as *n* → *∞*, where ||·|| denotes any matrix norm which is associated with the vector norm

(36) $$\begin{array}{c} ||\left(x,y\right)||=\sqrt{x^{2}+y^{2}}. \end{array}$$

**Proposition 11 (Perron's Theorem, [19])**

Suppose that condition (35) holds. If *X*_*n*_ is a solution of (34), then either *X*_*n*_ = 0 for all large *n* or

(37) $$\begin{array}{c} \rho =\underset{n\to \infty }{\lim}{\left(||X_{n}||\right)}^{1/n} \end{array}$$

exists and is equal to the modulus of one of the eigenvalues of matrix *A*.

**Proposition 12 (see [19])**

Suppose that condition (35) holds. If *X*_*n*_ is a solution of (34), then either *X*_*n*_ = 0  for all large *n* or

(38) $$\begin{array}{c} \rho =\underset{n\to \infty }{\lim}\frac{||X_{n+1}||}{||X_{n}||} \end{array}$$

exists and is equal to the modulus of one of the eigenvalues of matrix *A*.

Let {(*x*_*n*_, *y*_*n*_)} be an arbitrary solution of the system (2) such that $\lim_{n\to \infty }x_{n}=\bar{x}$ and $\lim_{n\to \infty }y_{n}=\bar{y}$ , where $\bar{x}\in \left[L_{1},U_{1}\right]$ and $\bar{y}\in \left[L_{2},U_{2}\right]$ . To find the error terms, one has from the system (2)

(39) xn+1−x¯=α1+β1xn−1a1+b1yn−α1+β1x¯a1+b1y¯=β1(xn−1−x¯)a1+b1yn−b1x¯(yn−y¯)a1+b1yn,yn+1−y¯=α2+β2yn−1a2+b2xn−α2+β2y¯a2+b2x¯=−b2y¯(xn−x¯)a2+b2xn+β2(yn−1−y¯)a2+b2xn.

Let $e_{n}^{1}=x_{n}-\bar{x}$ and $e_{n}^{2}=y_{n}-\bar{y}$ ; then one has

(40) $$\begin{array}{c} e_{n+1}^{1}=a_{n}e_{n-1}^{1}+b_{n}e_{n}^{2},\\ e_{n+1}^{2}=c_{n}e_{n}^{1}+d_{n}e_{n-1}^{2}, \end{array}$$

where

(41) $$\begin{array}{c} a_{n}=\frac{\beta_{1}}{a_{1}+b_{1}y_{n}},\;\;b_{n}=-\frac{b_{1}\bar{x}}{a_{1}+b_{1}y_{n}},\\ c_{n}=-\frac{b_{2}\bar{y}}{a_{2}+b_{2}x_{n}},\;\;d_{n}=\frac{\beta_{2}}{a_{2}+b_{2}x_{n}}. \end{array}$$

Moreover,

(42) $$\begin{array}{c} \underset{n\to \infty }{\lim}a_{n}=\frac{\beta_{1}}{a_{1}+b_{1}\bar{y}},\;\;\underset{n\to \infty }{\lim}b_{n}=-\frac{b_{1}\bar{x}}{a_{1}+b_{1}\bar{y}},\\ \underset{n\to \infty }{\lim}c_{n}=-\frac{b_{2}\bar{y}}{a_{2}+b_{2}\bar{x}},\;\;\underset{n\to \infty }{\lim}d_{n}=\frac{\beta_{2}}{a_{2}+b_{2}\bar{x}}. \end{array}$$

Now, the limiting system of error terms can be written as

(43) [en+11en+12en1en2] =(0−b1x¯a1+b1y¯β1a1+b1y¯0−b2y¯a2+b2x¯00β2a2+b2x¯10000100)  ×[en1en2en−11en−12],

which is similar to linearized system of (2) about the equilibrium point $\left(\bar{x},\bar{y}\right)$ . Using Proposition 11, one has following result.

**Theorem 13**

Assume that {(*x*_*n*_, *y*_*n*_)} is a positive solution of the system (2) such that $\lim_{n\to \infty }x_{n}=\bar{x}$ and $\lim_{n\to \infty }y_{n}=\bar{y}$ , where $\bar{x}\in \left[L_{1},U_{1}\right]$ and $\bar{y}\in \left[L_{2},U_{2}\right]$ . Then, the error vector $e_{n}=\left(\begin{array}{c} e_{n}^{1}\\ e_{n}^{2}\\ e_{n-1}^{1}\\ e_{n-1}^{2} \end{array}\right)$ of every solution of (2) satisfies both of the following asymptotic relations:

(44) $$\begin{array}{c} \underset{n\to \infty }{\lim}{\left(||e_{n}||\right)}^{1/n}=\left|\lambda_{1,2,3,4}F_{J}\left(\bar{x},\bar{y}\right)\right|,\\ \underset{n\to \infty }{\lim}\frac{||e_{n+1}||}{||e_{n}||}=\left|\lambda_{1,2,3,4}F_{J}\left(\bar{x},\bar{y}\right)\right|, \end{array}$$

where $\lambda_{1,2,3,4}F_{J}(\bar{x},\bar{y})$ are the characteristic roots of Jacobian matrix $F_{J}(\bar{x},\bar{y})$ .

## 5. Existence of Unbounded Solutions of (2)

In this section, we study the behavior of unbounded solutions of system (2).

**Theorem 14**

Consider system (2). Then, for every positive solution {(*x*_*n*_, *y*_*n*_)} of (2) the following statements are true:

- let *β*_1_ < *a*_1_ and *β*_2_ > *a*_2_ + *b*_2_ *U*_1_; then *y*_*n*_ → *∞* as *n* → *∞*;
- let *β*_2_ < *a*_2_ and *β*_1_ > *a*_1_ + *b*_1_ *U*_2_; then *x*_*n*_ → *∞* as *n* → *∞*.

**Proof**

(i) Suppose that *a*_1_ < *β*_1_; then it follows from Theorem 1 that *x*_*n*_ ≤ *α*_1_/(*a*_1_ − *β*_1_) = *U*_1_, *n* = 1,2,…. Furthermore, from system (2) it follows that

(45) yn+1=α2+β2yn−1a2+b2xn≥α2+β2yn−1a2+b2U1=c2+d2yn−1,

where

(46) $$\begin{array}{c} c_{2}=\frac{\alpha_{2}}{a_{2}+b_{2}U_{1}},\;\;d_{2}=\frac{\beta_{2}}{a_{2}+b_{2}U_{1}}. \end{array}$$

Consider the following second-order difference equation:

(47) $$\begin{array}{c} w_{n+1}=c_{2}+d_{2}w_{n-1},\;n=0,1,\ldots . \end{array}$$

The solution of (47) is given by

(48) $$\begin{array}{c} w_{n}=\frac{c_{2}}{1-d_{2}}+r_{1}d_{2}^{n/2}+r_{2}{\left(-\sqrt{d_{2}}\right)}^{n},\;n=1,2,\ldots , \end{array}$$

where *r*_1_,  *r*_2_ depend on initial values *w*_−1_, *w*_0_. Moreover, assume that *β*_2_ > *a*_2_ + *b*_2_ *U*_1_; that is, *d*_2_ = *β*_2_/(*a*_2_ + *b*_2_ *U*_1_) > 1; then we obtain that {*w*_*n*_} is divergent. Hence, by comparison, we have *y*_*n*_ → *∞* as *n* → *∞*.

(ii) Assume that *a*_2_ < *β*_2_; then from Theorem 1 we obtain that *y*_*n*_ ≤ *α*_2_/(*a*_2_ − *β*_2_) = *U*_2_,  *n* = 1,2,…. Moreover, from system (2) we have

(49) xn+1=α1+β1xn−1a1+b1yn≥α1+β1xn−1a1+b1U2=c1+d1xn−1,

where

(50) $$\begin{array}{c} c_{1}=\frac{\alpha_{1}}{a_{1}+b_{1}U_{2}},\;\;d_{1}=\frac{\beta_{1}}{a_{1}+b_{1}U_{2}}. \end{array}$$

Next, we consider the following second-order difference equation:

(51) $$\begin{array}{c} z_{n+1}=c_{1}+d_{1}z_{n-1},\;n=0,1,\ldots . \end{array}$$

Then, it is easy to see that solution of (51) is given by

(52) $$\begin{array}{c} z_{n}=\frac{c_{1}}{1-d_{1}}+r_{3}d_{1}^{n/2}+r_{4}{\left(-\sqrt{d_{1}}\right)}^{n},\;n=1,2,\ldots , \end{array}$$

where *r*_3_, *r*_4_ depend on initial values *z*_−1_, *z*_0_. Furthermore, suppose that *β*_1_ > *a*_1_ + *b*_1_ *U*_2_; that is, *d*_1_ = *β*_1_/(*a*_1_ + *b*_1_ *U*_2_) > 1; then one has {*z*_*n*_} that is divergent. Hence, by comparison we have *x*_*n*_ → *∞* as *n* → *∞*.

## 6. Periodicity Nature of Solutions of (2)

**Theorem 15**

Assume that *a*_1_ > *β*_1_ and *a*_2_ > *β*_2_; then system (2) has no prime period-two solutions.

**Proof**

On the contrary, suppose that the system (2) has a distinctive prime period-two solutions

(53) $$\begin{array}{c} \ldots ,\left(p_{1},q_{1}\right),\left(p_{2},q_{2}\right),\left(p_{1},q_{1}\right),\ldots \end{array}$$

where *p*_1_ ≠ *p*_2_, *q*_1_ ≠ *q*_2_, and *p*_*i*_,  *q*_*i*_ are positive real numbers for *i* ∈ {1,2}. Then, from system (2), one has

(54) $$\begin{array}{c} p_{1}=\frac{\alpha_{1}+\beta_{1}p_{1}}{a_{1}+b_{1}q_{2}},\;\;p_{2}=\frac{\alpha_{1}+\beta_{1}p_{2}}{a_{1}+b_{1}q_{1}},\\ q_{1}=\frac{\alpha_{2}+\beta_{2}q_{1}}{a_{2}+b_{2}p_{2}},\;\;q_{2}=\frac{\alpha_{2}+\beta_{2}q_{2}}{a_{2}+b_{2}p_{1}}. \end{array}$$

After some tedious calculations from (54), we obtain

(55) p1+p2=μ+4b2α1(a1−β1)(a2−β2)+μ2b2(a1−β1),p1p2=(μ+4b2α1(a1−β1)(a2−β2)+μ22b2(a1−β1))2,

(56) q1+q2=ν+4b2α1(a1−β1)(a2−β2)+ν2b1(a2−β2),q1q2=(ν+4b2α1(a1−β1)(a2−β2)+ν22b1(a2−β2))2,

where *μ* = (*a*_2_ − *β*_2_)(*β*_1_ − *a*_1_) + *b*_2_ *α*_1_ − *b*_1_ *α*_2_ and *ν* = (*a*_2_ − *β*_2_)(*β*_1_ − *a*_1_) − *b*_2_ *α*_1_ + *b*_1_ *α*_2_. From (55), it follows that

(57) $$\begin{array}{c} {\left(p_{1}+p_{2}\right)}^{2}-4p_{1}p_{2}=0. \end{array}$$

Similarly, from (56), we have

(58) $$\begin{array}{c} {\left(q_{1}+q_{2}\right)}^{2}-4q_{1}q_{2}=0. \end{array}$$

Obviously, from (57) and (58), one has *p*_1_ = *p*_2_ and *q*_1_ = *q*_2_, respectively, which is a contradiction. Hence, the proof is completed.

## 7. Examples

**Example 1**

Let *α*_1_ = 0.5, *β*_1_ = 12, *a*_1_ = 13, *b*_1_ = 0.2, *α*_2_ = 0.1, *β*_2_ = 17, *a*_2_ = 17.5, and *b*_2_ = 0.3. Then, system (2) can be written as

(59) $$\begin{array}{c} x_{n+1}=\frac{0.5+12x_{n-1}}{13+0.2y_{n}},\;\;y_{n+1}=\frac{0.1+17y_{n-1}}{17.5+0.3x_{n}}, \end{array}$$

with initial conditions *x*_0_ = 0.46, *x*_−1_ = 0.5, *y*_−1_ = 0.11, and *y*_0_ = 0.14.

**Figure 1:**
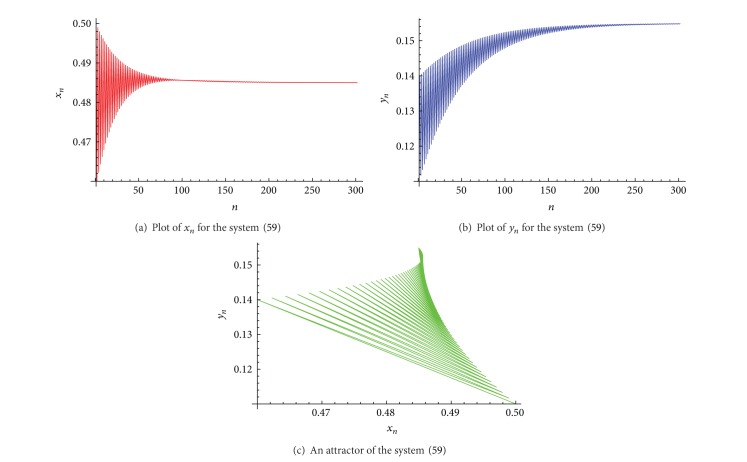
Plots for the system (59).

In this case, the unique positive equilibrium point of the system (59) is given by $\left(\bar{x},\bar{y})=(0.484974,0.154921\right)$ . Moreover, in Figure 1, the plot of *x*_*n*_ is shown in Figure 1(a), the plot of *y*_*n*_ is shown in Figure 1(b), and an attractor of the system (59) is shown in Figure 1(c).

**Example 2**

Let  *α*_1_ = 10,  *β*_1_ = 1.5,  *a*_1_ = 1.6,  *b*_1_ = 0.003,  *α*_2_ = 12,  *β*_2_ = 23,  *a*_2_ = 23.1, and  *b*_2_ = 0.02.  Then, system (2) can be written as

(60) $$\begin{array}{c} x_{n+1}=\frac{10+1.5x_{n-1}}{1.6+0.003y_{n}},\;\;y_{n+1}=\frac{12+23y_{n-1}}{23.1+0.02x_{n}}, \end{array}$$

with initial conditions *x*_−1_ = 82,  *x*_0_ = 89,  *y*_−1_ = 5.9, and  *y*_0_ = 6.

**Figure 2:**
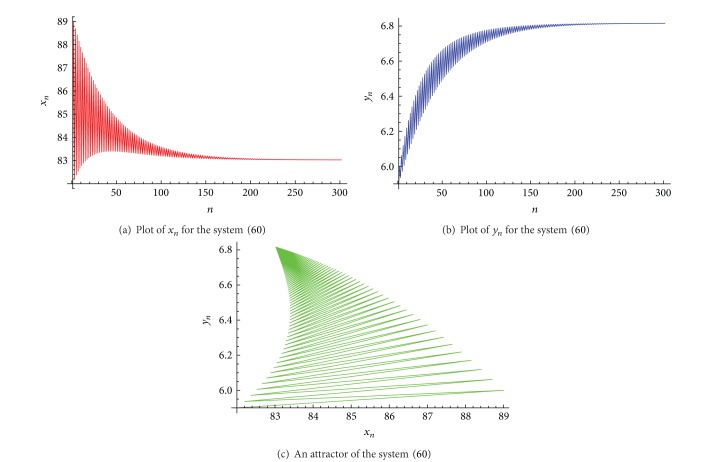
Plots for the system (60).

In this case, the unique positive equilibrium point of the system (60) is given by $\left(\bar{x},\bar{y})=(83.0225,6.81644\right)$ . Moreover, in Figure 2, the plot of *x*_*n*_ is shown in Figure 2(a), the plot of *y*_*n*_ is shown in Figure 2(b), and an attractor of the system (60) is shown in Figure 2(c).

**Example 3**

Let *α*_1_ = 3.2,  *β*_1_ = 8,  *a*_1_ = 8.1,  *b*_1_ = 5.5,  *α*_2_ = 4.2,  *β*_2_ = 16,  *a*_2_ = 16.1,  and *b*_2_ = 8.5. Then, system (2) can be written as

(61) $$\begin{array}{c} x_{n+1}=\frac{3.2+8x_{n-1}}{8.1+5.5y_{n}},\;\;y_{n+1}=\frac{4.2+16y_{n-1}}{16.1+8.5x_{n}}, \end{array}$$

with initial conditions *x*_−1_ = 3.9,  *x*_0_ = 3.5,  *y*_−1_ = 0.1, and  *y*_0_ = 0.12.

**Figure 3:**
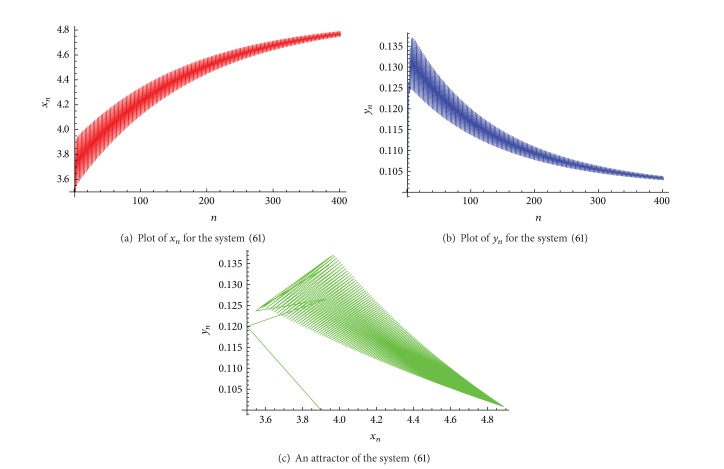
Plots for the system (61).

In this case, the unique positive equilibrium point of the system (61) is given by $\left(\bar{x},\bar{y})=(4.88876,0.10083\right)$ . Moreover, in Figure 3, the plot of *x*_*n*_ is shown in Figure 3(a), the plot of *y*_*n*_ is shown in Figure 3(b), and an attractor of the system (61) is shown in Figure 3(c).

## 8. Concluding Remarks

In literature, several articles are related to qualitative behavior of competitive system of planar rational difference equations [20]. It is very interesting mathematical problem to study the dynamics of competitive systems in higher dimension. This work is related to qualitative behavior of competitive system of second-order rational difference equations. We have investigated the existence and uniqueness of positive steady state of system (2). Under certain parametric conditions the boundedness and persistence of positive solutions is proved. Moreover, we have shown that unique positive equilibrium point of system (2) is locally as well as globally asymptotically stable. Furthermore, rate of convergence of positive solutions of (2) which converge to its unique positive equilibrium point is demonstrated. Finally, existence of unbounded solutions and periodicity nature of positive solutions of this competitive system are given.
